# Comparison of four different analyzers for prenatal trisomy 21 risk

**DOI:** 10.1515/almed-2025-0008

**Published:** 2025-06-04

**Authors:** Natalia García-Simón, Cristina Martínez-Payo, Francisco A. Bernabeu-Andreu, Encarnación Donoso-Navarro

**Affiliations:** Laboratory Medicine Department, Puerta de Hierro Majadahonda University Hospital, Majadahonda, Spain; Obstetrics and Gynecology Deparment, Puerta de Hierro Majadahonda University Hospital, Majadahonda, Spain

**Keywords:** first-trimester combined screening for trisomy 21, free beta subunit of the human chorionic gonadotropin, multiples of the median, pregnancy-associated plasma protein A, trisomy 21 false positive rate

## Abstract

**Objectives:**

Various analyzers are available for measuring first-trimester combined screening for trisomy 21 (T21) serum biomarkers (free-beta subunit of the human chorionic gonadotropin (β-hCG) and the pregnancy-associated plasma protein A (PAPP-A)). Our aim is to compare the analytical performance of four different analyzers and their false positive rates (FPR) for T21 risk.

**Methods:**

A 6-month study analyzed data from 1,136 pregnant women for first-trimester combined screening. Serum samples were processed using four analyzers (IMMULITE 2000, Centaur XP, Cobas e-411, KRYPTOR compact PLUS), each with its own risk calculation software. Comparative analyses of the biochemical markers and their multiples of the median (MoMs) were conducted using Passing-Bablok and Bland-Altman methods.

**Results:**

Significant variability was observed among the analyzers. For free β-hCG, only Centaur XP and KRYPTOR compact PLUS were interchangeable. For PAPP-A, only IMMULITE 2000 and Cobas e-411 were comparable. However, no analyzer pair was interchangeable for both markers simultaneously. Free β-hCG multiples of the median (MoMs) were highest in IMMULITE 2000 (1.85 MoM (IQR: 1.4–2.97)) and lowest in KRYPTOR compact PLUS (1.5 MoM (IQR: 1.23–2.21)). PAPP-A MoMs were lowest in IMMULITE 2000 (0.52 MoM (IQR: 0.38–0.82)) and highest in Cobas e-411 (0.58 MoM (IQR: 0.39–0.90)). In risk assessment, all analyzers detected true T21 cases but varied in their FPR, with Centaur XP (3.8 %), Cobas e-411 (2.5 %) and KRYPTOR compact PLUS (2.3 %) showing a FPR below 5 %.

**Conclusions:**

Measurement of serum biomarkers by the four analyzers is not interchangeable. KRYPTOR compact PLUS showed the lowest FPR for risk assessment.

## Introduction

First-trimester combined screening for trisomy 21 (T21) risk assessment, validated in 2009 by Nicolaides et al. [1], has been widely used in obstetrics consultations [2], [3], [4]. This screening employs an algorithm that incorporates both serum and ultrasound parameters. Serum biomarkers encompass the free beta subunit of the human chorionic gonadotropin (β-hCG) and the pregnancy-associated plasma protein A (PAPP-A), while the ultrasound parameter corresponds to nuchal translucency (NT) thickness [5]. These parameters refer to the gestational age, determined by the ultrasound measurement of the embryo’s crown-rump length.

They adjust the initial risk of the pregnancy being affected by trisomy 21, which is based on the mother’s age-related risk. Based on this analysis, patients can be categorized as low and high risk.

While the cell-free fetal DNA (cffDNA) screening test has demonstrated a better detection rate compared to serum screening tests [6], it is a costly technique and may not always be available, with cost-effectiveness generally acceptable only when offered to high-risk patients [7]. Consequently, first-trimester combined screening continues to be considered the primary option for all pregnant women [7], [8], [9], [10], [11], [12], employing cffDNA testing selectively to minimize invasive procedures in cases of positive results.

Different approaches for managing patients based on their risk level are available, although guidelines concur with offering confirmation through diagnostic testing (chorionic villus sampling (CVS) and amniocentesis) to those identified as high risk [7], [8], [9], [10], [11]. The downside of these invasive tests is that they entail serious complications, such as miscarries and infections, so it is crucial that the initial screening test offers minimum false positives rate (FPR) maintaining a high detection rate. First-trimester combined screening has shown to have a detection rate of 80–90 %, for a FPR of 5 % [2], 9], 13], 14].

Nowadays, there are various automatized analyzers available for measuring serum free β-hCG and PAPP-A, each one equipped with its own software for the calculation of fetal aneuploidy risk. These analytical systems utilize serum biomarkers, nuchal translucency (NT) thickness, and maternal age – which serves as the basis for background T21 risk – along with additional parameters that may influence risk. These factors include gestational age, maternal weight, ethnicity, tobacco use, mode of conception (natural or assisted), and the number of fetuses [13], [15], [16], [17], [18]. All these variables are intrinsic of the patient, and therefore independent of the system used, except for serum biomarkers as each instrument has its own analytical variability. This discrepancy among analyzers is mitigated in the risk calculation by expressing free β-hCG and PAPP-A analytes as multiples of the median (MoM) rather than concentration units [19]. These MoMs are the deviation of a median calculated from a reference population for each gestational age. However, the population database utilized as reference is not the same for all systems, varying in origin, size and composition [20], and sometimes are undisclosed to the user.

This variability among systems can result in significant disparities in the risk outcome [21], 22] denoting a substantial concern as the same pregnant women could have different risk results depending on the instrument employed. Despite this, there are very few comparative studies among different analyzers and their respective risk assessment with discrepant results [22], [23], [24], [25]. To our knowledge, this is the first study to compare analytical performance of four different analyzers and their FPR for first-trimester combined screening for T21.

## Materials and methods

### Subjects

During a 6-month period (from February to July 2020), data were collected from 1,136 pregnant women who were referred to Puerta de Hierro Majadahonda University Hospital (Spain) for first-trimester combined screening. Serum samples were collected between 9th + 1 week and 13th+ 3 week of gestation, and NT thickness was measured between 10th + 6 week and 13th + 6 week of gestation [26].

Following the current guidelines in our autonomous community (Madrid) [27], the first-trimester combined screening was performed sequentially, with the serum sample and NT thickness measurement taken during different weeks of gestation. Risk below 1/270 were considered as low risk of having a fetus with T21 while those equal to or above 1/270 were categorized as high risk. Patients identified as high risk were initially offered cffDNA testing, and if the results confirmed the elevated risk, they were subsequently referred for invasive testing (chorionic villus sampling (CVS) or amniocentesis) to confirm the diagnosis. In case of very high risk (above 1/10), NT>99th percentile, or ultrasound malformation, a direct invasive test would have been recommended without prior cffDNA testing. However, none of the patients met these criteria.

For the calculation of the false positive rate (FPR), the gold standard was established based on either the birth outcome, the genetic analysis following diagnostic testing (CVS or amniocentesis), or the results of a post-mortem genetic study in cases of abortion. Consequently, individuals who were born without T21, those with a negative T21 diagnostic test, or those who tested negative for T21 in a post-mortem study were classified as true negatives. On the other hand, individuals born with T21, those with a positive T21 diagnostic test, or those with a T21-positive result in a post-mortem study were classified as true positives.

The study was granted approval by the Ethics Committee of Puerta de Hierro Majadahonda Hospital (PI_13–20). Prior to testing, participants received medical counseling and provided clinical consent for genetic testing.

### Samples

Initially, all 1,136 serum samples were processed using the routine analyzer, IMMULITE 2000 (Siemens Healthinneers, Erlangen, Germany) for clinical assessment. Subsequently, they were preserved by storing at −80 °C for 6 months. From this analysis, 63 samples yielded a risk result of ≥1/270 for T21 thereby defining the high-risk group. The low-risk group consisted of 50 patients randomly selected from the pool of 1,073 individuals with a risk result of <1/270. However, risk assessment was unattainable for five of those patients due to insufficient echography data, resulting in a final count of low-risk patients of 45. The total number of patients included in this study was n=108, divided in high-risk (n=63) and low-risk (n=45) groups. After the selection of study samples, they were thawed at room temperature and homogenized by vortex.

### Instruments

Comparative study was conducted involving four distinct instruments: IMMULITE 2000, Centaur XP (Siemens Healthinneers), Cobas e-411 (Roche, Basel, Switzerland) and KRYPTOR compact PLUS (Thermo Fisher Scientific, Massachusetts, USA). Both IMMULITE 2000 and Centaur XP utilize chemiluminescent immunoassays, while the Cobas e-411 employs an electrochemiluminescence immunoassay (ECLIA). In contrast, the KRYPTOR Compact PLUS uses an automated immunofluorescent assay based on TRACE technology (time-resolved amplified cryptate emission).

Each analyzer’s risk assessment was calculated using its corresponding software: PRISCA for IMMULITE 2000 and Centaur XP, SsdwLab6 for Cobas e-411 and Thermo Scientific Fast Screen™ for KRYPTOR compact PLUS.

To calculate the free β-hCG MoMs and PAPP-A MoMs, the PRISCA software utilizes data from a cohort of 842 Caucasian pregnant women in the first trimester, collected from routine pregnancy screenings at the Fetal Medicine Research Institute in the United Kingdom. Median values for β-hCG and PAPP-A were determined at each completed week of gestation during the first trimester. Twin pregnancies and smokers were excluded from the database before calculating the medians. After this, MoMs are periodically recalculated using data from ongoing laboratory screenings. At the time of this study, MoMs were calculated based on historical data from the laboratory.

For the SsdwLab6 software, MoMs were calculated using data from a study involving 4,746 first-trimester free β-hCG and PAPP-A values (gestational weeks 8 + 0 to 13 + 6). Median values were estimated for each day of the respective gestational age by regression of the calculated medians per day [28].

Fast Screen™ used the medians derived from a study by Wright et al., which involved serum samples from 222,475 pregnant women, including 886 pregnancies with trisomy 21 [26].

Each program utilized identical datasets, including free β-hCG and PAPP-A values along with precise gestational age at sampling, NT thickness and its corresponding gestational age, maternal age, maternal weight, smoking status, presence of diabetes, ethnicity, type of conception (natural vs. *in vitro*), and number of fetuses. All parameters remained constant except for the free β-hCG and PAPP-A values, which varied based on the instrument used and the reference medians for each program. All four methods standardized the free β-hCG analyte against the International Reference Preparation of Chorionic Gonadotropin β subunit from the National Institute for Biological Standards and Control (NIBSC), code 75/551. In contrast, the PAPP-A analyte was standardized against the WHO standard preparation IRP 78/610 for the Cobas e-411 and KRYPTOR Compact PLUS. For the IMMULITE 2000 and Centaur XP, PAPP-A was standardized using an internal standard, manufactured with qualified materials and measurement procedures, as indicated in the technique inserts.

### Statistics

The biochemical serum marker results were compared both in terms of concentration and MoMs across analyzers using Passing-Bablok and Bland-Altman analyses, following CLSI EP09-A3 recommendations [29]. In Passing-Bablok regression, analyzers were considered to be non-interchangeable if one or more of the following criteria were met: 95 % confidence interval (95% CI) of intercept did not include value zero (0) and/or 95% CI of slope did not include value one (1) [30]. In the Bland-Altman analysis, significant bias was identified when the line of equality (0) did not fall in the 95%CI of the mean difference (p<0.05) [31]. Instruments and risk programs were considered interchangeable only if both Passing-Bablok and Bland-Altman analyses revealed no significant bias. Shapiro-Wilk test was used to check normality. A p-value <0.05 was considered statistically significant.

Statistical programs used was MedCalc Statistical Software version 18.2.1 (MedCalc Software bvba, Ostend, Belgium, http://www.medcalc.org; 2018).

## Results

### Biomarkers comparison

Concentrations of free β-hCG and PAPP-A were expressed in ng/mL and mIU/mL respectively. In the analysis of free β-hCG, regression comparisons for analyzer performance revealed very high Spearman correlation coefficients (ρ) exceeding 0.97 in all cases. However, except in the case of Centaur XP when compared to KRYPTOR compact PLUS and to Cobas e-411, all the rest showed a proportional error since none of them met the required criterion for slope. Additionally, Cobas e-411 exhibited a constant error when compared with Centaur XP and with KRYPTOR compact PLUS, but not with IMMULITE 2000. Bland-Altman analysis indicated significant bias (p<0.001) in all comparisons except for Centaur XP vs. KRYPTOR compact PLUS (p=0.389). Thus, only Centaur XP and KRYPTOR compact PLUS revealed to be interchangeable in both Passing-Bablok and Bland-Altman analyses.

Regarding PAPP-A measurement, regression analysis showed that the only acceptable comparison was between IMMULITE 2000 and Cobas e-411, while all other comparisons exhibited constant error. Similarly, these two analyzers were the only ones that did not exhibit significant differences in PAPP-A measurement in the Bland-Altman analysis (p=0.836).

Detailed results of the comparisons among the different analyzers are presented in Table 1. Scatter diagrams illustrating Passing-Bablok analyses for each study are depicted in Supplemental Figure 1 and Supplemental Figure 2 for free β-hCG and PAPP-A, respectively, while Bland-Altman analyses are shown in Supplemental Figure 3 and Supplemental Figure 4.

**Table 1:** Comparison of free β-hCG and PAPP-A levels among the different analyzers.

Analyzers	Analyte	ρ	Intercept (95 % CI)	Slope (95 % CI)	Mean (95 % CI)	p-Value
IMMULITE 2000 vs. Centaur XP	Free β-hCG	0.988	0.17 (−1.63 to 1.53)	0.87 (0.84–0.90)^a^	15.9 (10.9 to 20.8)^a^	<0.001
	PAPP-A	0.979	0.02 (−0.03 to 0.06)	1.48 (1.42–1.54)^a^	−0.69 (−0.8 to −0.58)^a^	<0.001
IMMULITE 2000 vs. KRYPTOR compact PLUS	Free β-hCG	0.981	2.12 (−0.33 to 3.71)	0.85 (0.82–0.89) ^a^	17.3 (13.1 to 21.6)^a^	<0.001
	PAPP-A	0.978	0.004 (−0.03 to 0.04)	1.21 (1.18–1.25)^a^	−0.34 (−0.41 to −0.28)^a^	<0.001
IMMULITE 2000 vs. Cobas e-411	Free β-hCG	0.978	−1.63 (−4.05 to 0.40)	0.83 (0.79–0.86)^a^	20.7 (16.5 to 24.9)^a^	<0.001
	PAPP-A	0.973	0.03 (−0.01 to 0.07)	0.96 (0.92–1.01)	−0.005 (−0.05 to 0.04)	0.836
Centaur XP vs. KRYPTOR compact PLUS	Free β-hCG	0.985	1.32 (−0.74 to 3.45)	0.97 (0.94–1.02)	1.17 (−1.51 to 3.86)	0.389
	PAPP-A	0.994	0.02 (−0.01 to 0.03)	1.21 (1.18–1.24)^a^	0.35 (0.28 to 0.4)^a^	<0.001
Centaur XP vs. Cobas e-411	Free β-hCG	0.98	−2.3 (−4.78 to −0.76)^a^	0.97 (0.93–1.01)	5.94 (3.75 to 8.13)^a^	<0.001
	PAPP-A	0.988	0.03 (−0.01 to 0.05)	0.66 (0.64–0.69)^a^	0.69 (0.57 to 0.8)^a^	<0.001
KRYPTOR compact PLUS vs. Cobas e-411	Free β-hCG	0.969	3.52 (1.21 to 6.06)^a^	1.02 (0.97–1.07)	4.83 (2.23 to 7.53)^a^	<0.001
	PAPP-A	0.989	0.04 (0.01 to 0.67)^a^	0.79 (0.76–0.81)^a^	0.34 (0.27 to 0.41)^a^	<0.001

ρ, Spearman correlation coefficient; 95 % CI, 95 % confidence interval. ^a^Criteria for acceptable interchangeability not met.

### MoMs comparison

The ρ coefficients were generally lower compared to those obtained in the comparison of analytes. Contrary to the analytes’ comparison, MoMs comparison did not demonstrate interchangeability for free β-hCG MoMs for none of the instruments, all of which exhibited bias in one or both statistical analyses. Only IMMULITE 2000 and Cobas e-411 had an acceptable result in Passing-Bablok analysis, but not in the Bland-Altman analysis. All comparisons of free β-hCG MoMs showed significant differences in the Bland-Altman analysis, except for Centaur XP and Cobas e-411, which, conversely, displayed bias in the Passing-Bablok regression. The median values of free β-hCG MoMs for the different instruments were as follows: 1.85 MoM (IQR: 1.4–2.97) for IMMULITE 2000, 1.75 MoM (IQR: 1.28–2.54) for Centaur XP, 1.75 MoM (IQR: 1.27–2.62) for Cobas e-411, and 1.5 MoM (IQR: 1.23–2.21) for KRYPTOR compact PLUS.

Regression analysis of PAPP-A MoMs across different pairs of instruments revealed that all met the required criteria except for IMMULITE 2000 vs. KRYPTOR compact PLUS, which exhibited constant bias. Bland-Altman analysis showed a p<0.001 for all comparisons involving IMMULITE 2000. Conversely, the remaining analyzers did not demonstrate statistical significance, with p-values of 0.96 for Centaur XP vs. KRYPTOR compact PLUS, 0.09 for Centaur XP vs. Cobas e-411, and 0.24 for KRYPTOR compact PLUS vs. Cobas e-411. Regarding medians, the median value of PAPP-A MoMs for IMMULITE 2000 was 0.52 MoM (IQR: 0.38–0.82), 0.55 MoM (IQR: 0.41–0.91) for Centaur XP, 0.54 MoM (IQR: 0.42–0.84) for KRYPTOR compact PLUS, and 0.58 MoM (IQR: 0.39–0.90) for Cobas e-411.

All results are summarized in Table 2. The scatter diagrams for Passing-Bablok analysis of free β-hCG and PAPP-A are presented in Supplemental Figure 5 and Supplemental Figure 6, respectively, while those for Bland-Altman analysis are shown in Supplemental Figure 7 and Supplemental Figure 8.

**Table 2:** Comparison of free β-hCG MoMs and PAPP-A MoMs among the different programs.

Programs	MoM	ρ	Intercept (95 % CI)	Slope (95 % CI)	Mean (95 % CI)	p-Value
IMMULITE 2000 vs. Centaur XP	Free β-hCG	0.973	0.11 (0.01 to 0.19)^a^	0.87 (0.82–0.92)^a^	0.19 (0.11 to 0.29)^a^	<0.001
	PAPP-A	0.957	0.04 (−0.02 to 0.06)	1.04 (0.98–1.13)	−0.09 (−0.13 to −0.05)^a^	<0.001
IMMULITE 2000 vs. KRYPTOR compact PLUS	Free β-hCG	0.901	0.03 (−0.09 to 0.16)	0.81 (0.75–0.88)^a^	0.43 (0.31 to 0.55)^a^	<0.001
	PAPP-A	0.935	0.03 (0.002 to 0.08)^a^	1.02 (0.92–1.08)	−0.08 (−0.13 to −0.04)^a^	<0.001
IMMULITE 2000 vs. Cobas e-411	Free β-hCG	0.91	−0.05 (−0.25 to 0.81)	0.98 (0.89–1.05)	0.24 (0.09 to 0.38)^a^	0.002
	PAPP-A	0.882	−0.01 (−0.06 to 0.04)	1.03 (0.95–1.13)	−0.06 (−0.10 to −0.01)^a^	0.015
Centaur XP vs. KRYPTOR compact PLUS	Free β-hCG	0.969	−0.003 (−0.1 to 0.12)	0.9 (0.84–0.97)^a^	0.19 (0.11 to 0.27)^a^	<0.001
	PAPP-A	0.952	0.01 (−0.03 to 0.04)	1.01 (0.94–1.08)	0.0 (−0.03 to 0.03)	0.959
Centaur XP vs. Cobas e-411	Free β-hCG	0.958	−0.19 (−0.37 to −0.03)^a^	1.13 (1.02–1.23)^a^	−0.01 (−0.13 to 0.12)	0.924
	PAPP-A	0.973	−0.01 (−0.04 to 0.02)	0.95 (0.9–1.01)	0.03 (−0.004 to 0.06)	0.085
KRYPTOR compact PLUS vs. Cobas e-411	Free β-hCG	0.947	−0.13 (−0.31 to −0.001)^a^	1.2 (1.09–1.32)^a^	−0.19 (−0.31 to −0.08)^a^	0.002
	PAPP-A	0.947	−0.02 (−0.09 to 0.03)	1 (0.91–1.12)	0.03 (−0.02 to 0.07)	0.235

MoM, multiples of the median; ρ, Spearman correlation coefficient; 95 % CI, 95 % confidence interval. ^a^Criteria for acceptable interchangeability not met.

### Risk results

All patients with a risk ≥1/270 (n=63) (determined by IMMULITE 2000) were referred to cffDNA analysis. Among them, 58 resulted in low-risk, out of which 56 had normal birth, including one patient who gave birth to twins. Two of these cffDNA low-risk patients suffered spontaneous abortion, and postmortem DNA analysis revealed no abnormalities. Of the remaining five patients with high risk, cffDNA analysis showed T21 in all cases. After this confirmation, an invasive technique was performed in all five cases (3 amniocentesis and 2 CVS), leading to the diagnosis of T21 in four of them. Subsequently, the mothers opted for pregnancy termination. In the fifth patient, CVS showed a normal genetic result, but she experienced a miscarriage due to the invasive procedure. Notably, this patient’s sample also yielded a high-risk result on the three other analyzers and would have been referred to invasive technique regardless. Patients with a T21 risk under 1/270 all delivered euploid children, except for one case which resulted in a spontaneous abortion. DNA analysis performed to the fetus showed no evidence of trisomy.

In terms of accuracy, all analyzers correctly identified the four cases of T21 as high risk, resulting in a 0 % false negative rate in our study cohort for all of them. Regarding false positive results, IMMULITE 2000 identified 63 patients out of the total 1,137 analyzed as high risk. Among these, four were true positives, leaving 59 cases as false positives, resulting in a false positive rate (FPR) of 5.2 % (59/1,137). Centaur XP exhibited a false positive rate of 3.8 % (43/1,137), Cobas e-411 showed a false positive rate of 2.5 % (28/1,137), and KRYPTOR compact PLUS had a false positive rate of 2.3 % (26/1,137).

## Discussion

Screening for trisomy 21 in pregnant women is widely implemented and employs various approaches. In recent years, cffDNA testing has demonstrated superior results, but its current cost limits its use to pregnant women at high risk of T21 [6], 7]. In terms of serum screening, the most prevalent method is first-trimester combined screening, which calculates T21 risk using both clinical (NT thickness) and biochemical parameters (free β-hCG and PAPP-A) [2]. To account for the variability of biochemical analytes across different gestational ages, the values are converted into multiples of the median (MoMs) to calculate the T21 risk. There are several instruments that measure these analytes, each one using its own calculated MoMs. Therefore, our study aimed to assess the comparability among different analyzers for the measurement of free β-hCG and PAPP-A and their MoMs.

Comparison of the biochemical markers revealed that results were solely comparable between Centaur XP and KRYPTOR compact PLUS for free β-hCG, and IMMULITE 2000 and Cobas e-411 for PAPP-A. However, none of the instruments were comparable for both analytes simultaneously. As both analytes are necessary for risk calculation, we concluded that no single analyzer could replace another for the measurement of free β-hCG and PAPP-A.

Regarding MoMs, IMMULITE 2000 yielded higher free β-hCG MoM values compared to the other instruments. Centaur XP and Cobas e-411 exhibited similar results for free β-hCG MoMs, while KRYPTOR compact PLUS showed the lowest values. For PAPP-A MoMs, IMMULITE 2000 produced the lowest values, while Cobas e-411 showed the highest. Centaur XP and KRYPTOR compact PLUS demonstrated similar intermediate medians for PAPP-A MoMs. Engell et al. [23] compared MoMs of free β-hCG and PAPP-A between Cobas e-411 and KYRPTOR and concluded that Cobas e-411 values were lower than KRYPTOR compact PLUS’s. However, Hörmansdörfer et al. [24] had lower results in KRYPTOR compact PLUS’ PAPP-A MoMs than in Cobas e-411, but similar results for free β-hCG MoMs for both instruments. Our findings supported those of the latter, as our results showed that PAPP-A MoMs values for Cobas e-411 were higher than KRYPTOR compact PLUS’. For free β-hCG MoMs where we found a median difference of 0.2 MoM between the two instruments, whereas Hörmansdörfer et al. only reported a 0.02 MoM difference. Furthermore, other study [25] compared Cobas e-411 with IMMULITE 2000 and stated that there was a significant correlation between them, a conclusion we could not replicate. Passing-Bablok and Bland Altman studies revealed that only the comparison of PAPP-A MoMs between Centaur XP and KRYPTOR compact PLUS, and between Centaur XP and Cobas e-411 exhibited no bias. Nevertheless, these findings did not extend to free β-hCG MoMs, which presented significant bias in all analyzer comparisons. Consequently, we concluded that no interchangeability could be warranted, and thus, each analyzer should employ its own risk calculation program.

The most significant distinction among analyzers, considering the number of tested cases, lied in their false positive rate. For risk results, all four programs detected all positive cases but only Centaur XP, Cobas e-411 and KRYPTOR compact PLUS achieved a suitably FPR under 5 % [32], [33], [34], [35], with KRYPTOR compact PLUS having the lowest rate. Interestingly, despite IMMULITE 2000 and Centaur XP utilizing the same risk calculation program and MoMs, they exhibited different FPR (5.2 vs. 3.8 %, respectively), indicating differences in analyte measurement. Another comparison between KRYPTOR compact PLUS and IMMULITE 2000 concluded better performance by KRYPTOR compact PLUS, consistent with our findings [22]. Additionally, Engell et al. [23] reported variations in MoMs but found no differences in overall performance between KRYPTOR compact PLUS and Cobas e-411, being 5.1 % for both. However, discrepancies between our and their FPR may be attributed to differences in risk assessment software. The variations in MoMs among analyzers underline the differences in the medians utilized by each program for calculation. These discrepancies arise from the population data used to establish those medians, which, in some cases, are unknown to the user. While we opted to use the program associated with each analyzer, provided by ThermoFisher and Roche, respectively, Engell et al. [23] used the same program (Astraia, Gmbh, Munich, Germany) for both instruments which did not correspond to that provided by either of the commercial brands.

The main limitation of this study is the relatively low number of true positives. This comparative study was conducted during a period of transition in laboratory instruments. As we were required to select the new platform that best suited our needs within a limited timeframe, extending the sample collection period was not feasible and, as a result, we were unable to gather additional true positive cases. Furthermore, given this situation of internal competition, none of the instruments were subjected to an external control.

In terms of cffDNA, of all high-risk patients identified through first-trimester combined screening, cffDNA correctly identified all high-risk cases confirmed later by an invasive technique. However, in one case, cffDNA showed T21 that was not later corroborated by CVS. In the remaining cases, cffDNA results were negative, confirmed by normal newborn birth. These findings underscore the high, although not infallible, accuracy of cffDNA test. Despite this, first-trimester combined screening for T21 remains the preferred approach in Spain due to its broader accessibility. Although cfDNA testing is available, is a costly technique, making it essential to maintain a low false positive rate. Failing to do so could result in unnecessary increases in healthcare costs, placing an additional strain on the system through the need for more confirmatory tests and follow-up procedures. This not only compromises the system’s efficiency but also adds significant pressure on patients, who would receive a provisional pathological result, leading to considerable anxiety and emotional distress.

In conclusion, IMMULITE 2000, Centaur XP, KRYPTOR compact PLUS, and Cobas e-411 are not interchangeable. Therefore, if there is a substitution of the laboratory analyzer used in first-trimester combined screening for T21, it must be mandatorily accompanied by its own risk program, and the change should be effectively communicated to the clinical staff. Moreover, one of the main considerations when selecting an analyzer is its false positive rate to minimize unnecessary referrals to invasive techniques.

## Supplementary Material

Supplementary Material

Supplementary Material

Supplementary Material

Supplementary Material

Supplementary Material

Supplementary Material

Supplementary Material

Supplementary Material
