# Supplementary figures and images for: Comparison of four different analyzers for prenatal trisomy 21 risk
Source: Adv Lab Med. 2025 Jun 4;6(4):411–8. doi: 10.1515/almed-2025-0008 (PMC12744378; doi:10.1515/almed-2025-0008)

**Supplemental Figure 1.** Passing-Bablok comparison scatter diagrams for free β-hCG levels.


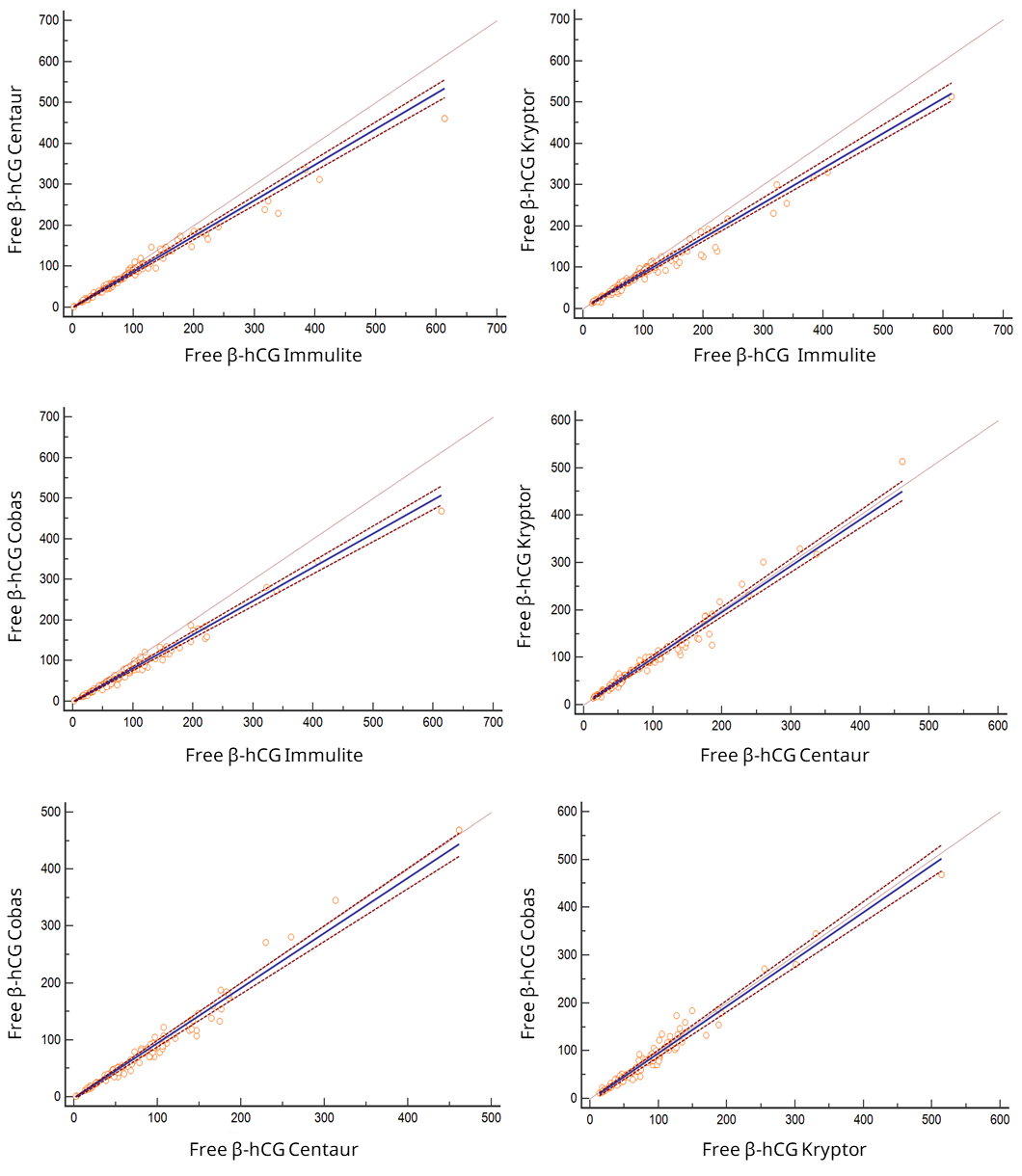

Supplement: Supplementary file 1 — Supplementary Material [file j_almed-2025-0008_suppl_001.docx]

**Supplemental Figure 2.** Passing-Bablok comparison scatter diagrams for PAPP-A levels.


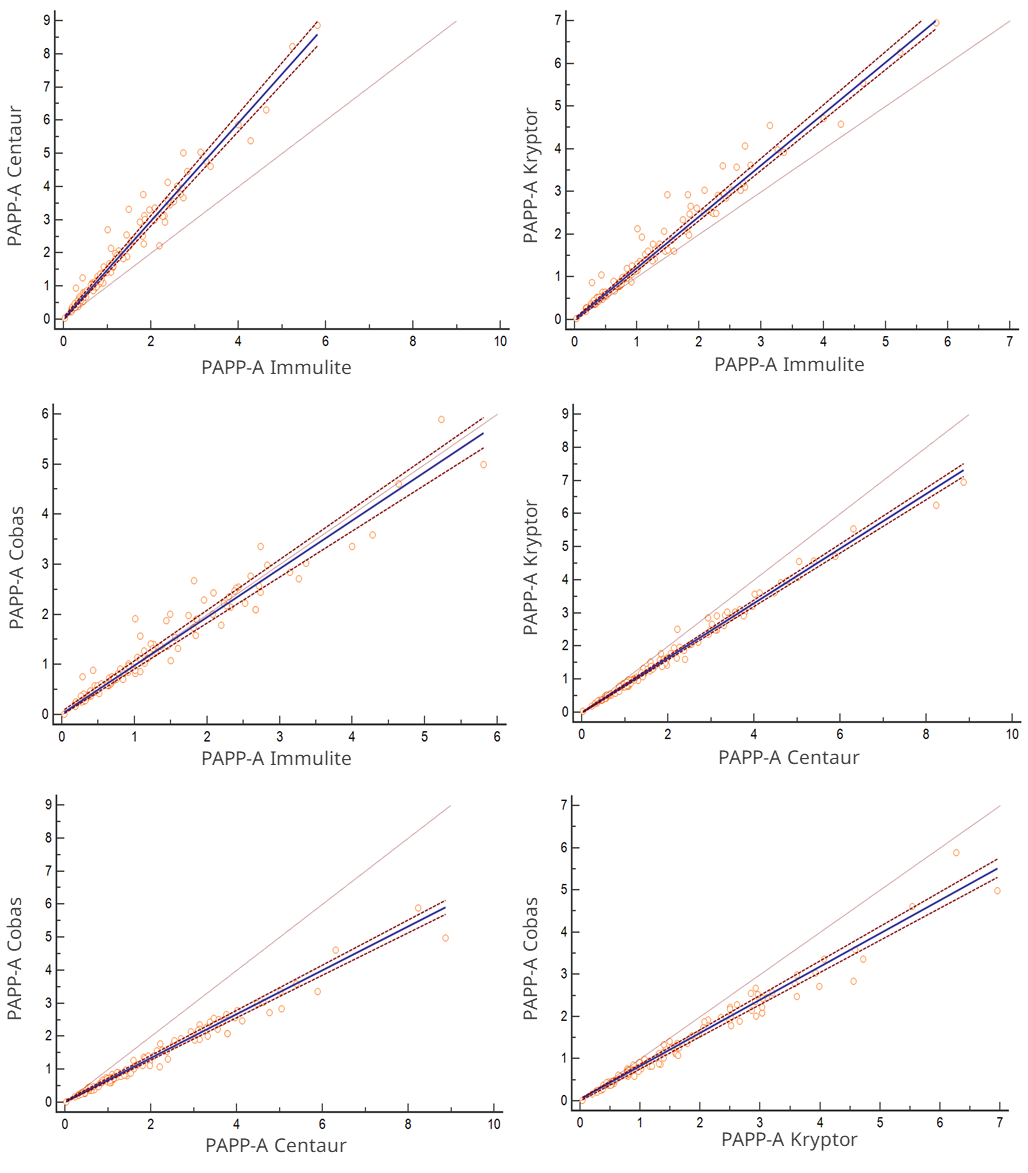

Supplement: Supplementary file 2 — Supplementary Material [file j_almed-2025-0008_suppl_002.docx]

**Supplemental Figure 3**. Bland-Altman comparison scatter diagrams for free β-hCG levels.


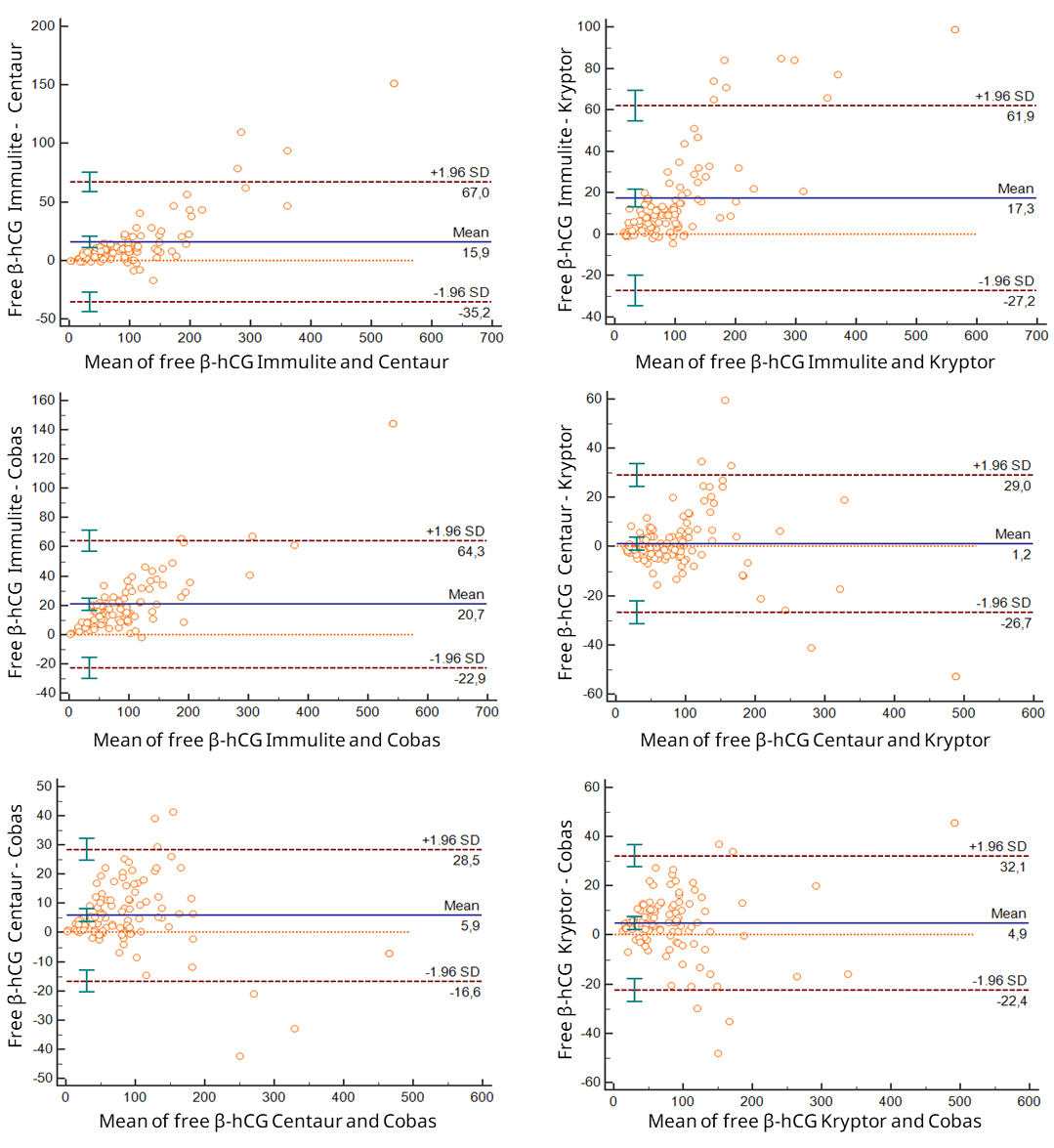

Supplement: Supplementary file 3 — Supplementary Material [file j_almed-2025-0008_suppl_003.docx]

**Supplemental Figure 4.** Bland-Altman comparison scatter diagrams for PAPP-A levels.


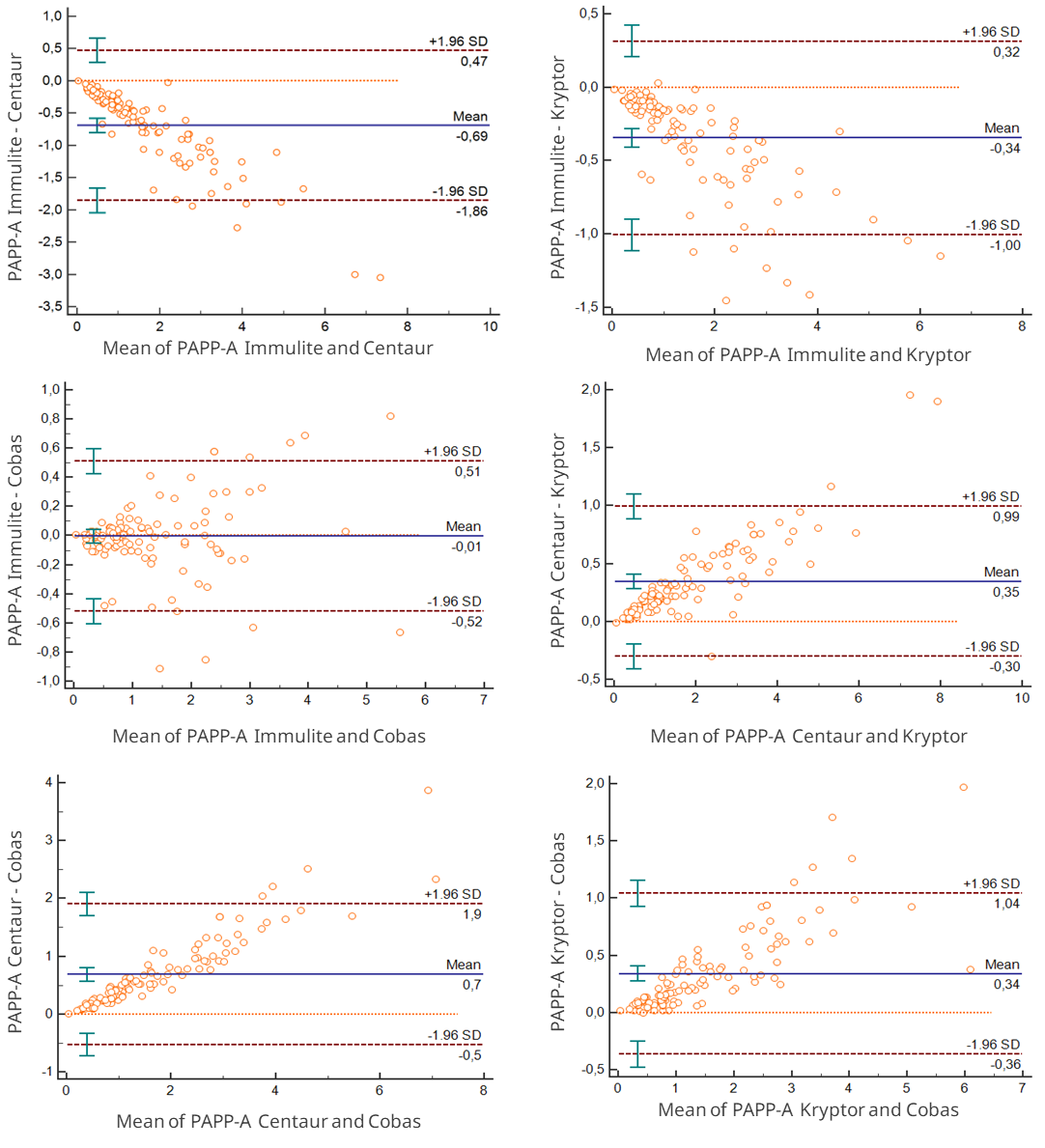

Supplement: Supplementary file 4 — Supplementary Material [file j_almed-2025-0008_suppl_004.docx]

**Supplemental Figure 5.** Passing-Bablok comparison scatter diagrams for free β-hCG MoM.


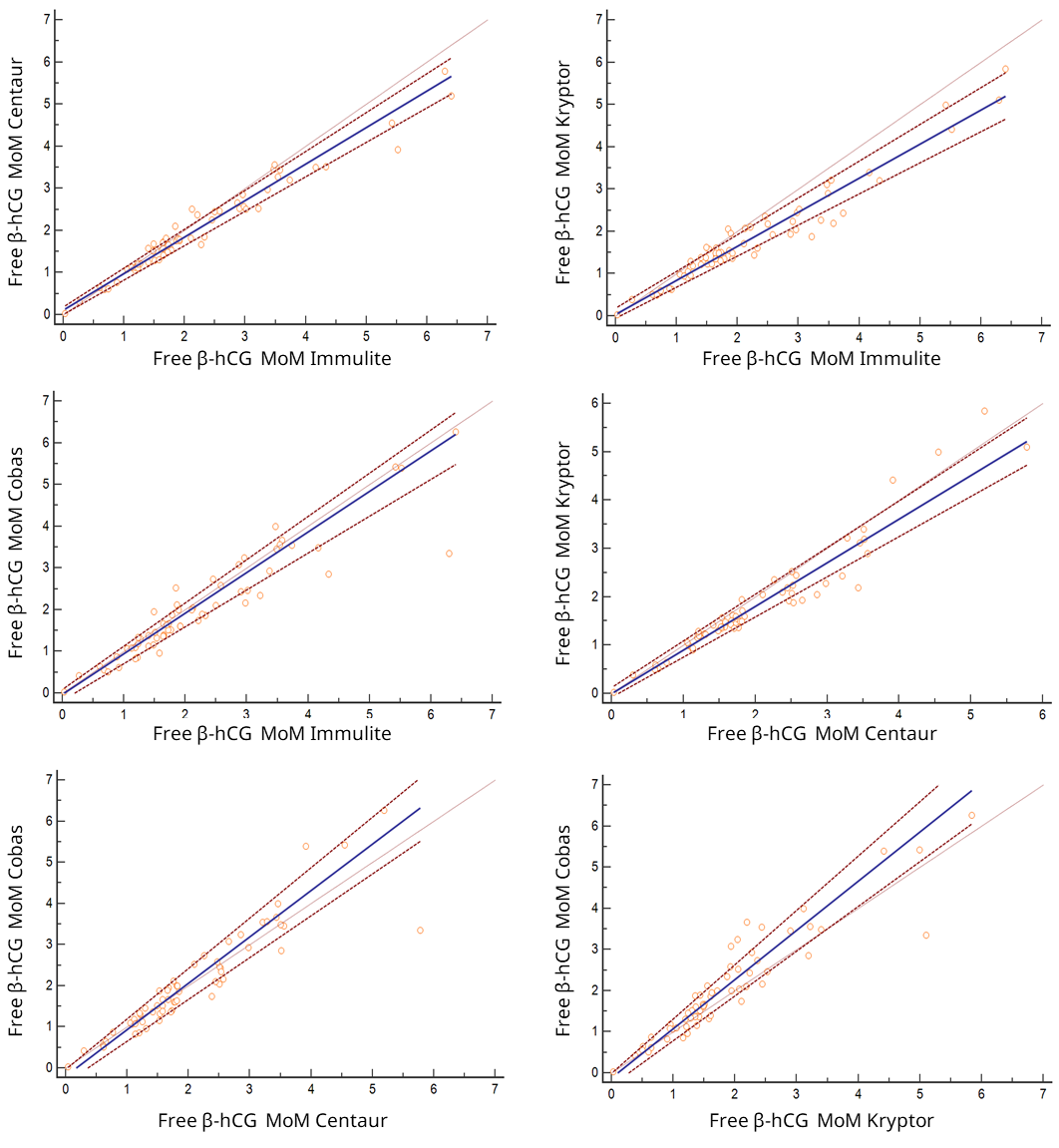

Supplement: Supplementary file 5 — Supplementary Material [file j_almed-2025-0008_suppl_005.docx]

**Supplemental Figure 6**. Passing-Bablok comparison scatter diagrams for PAPP-A MoM


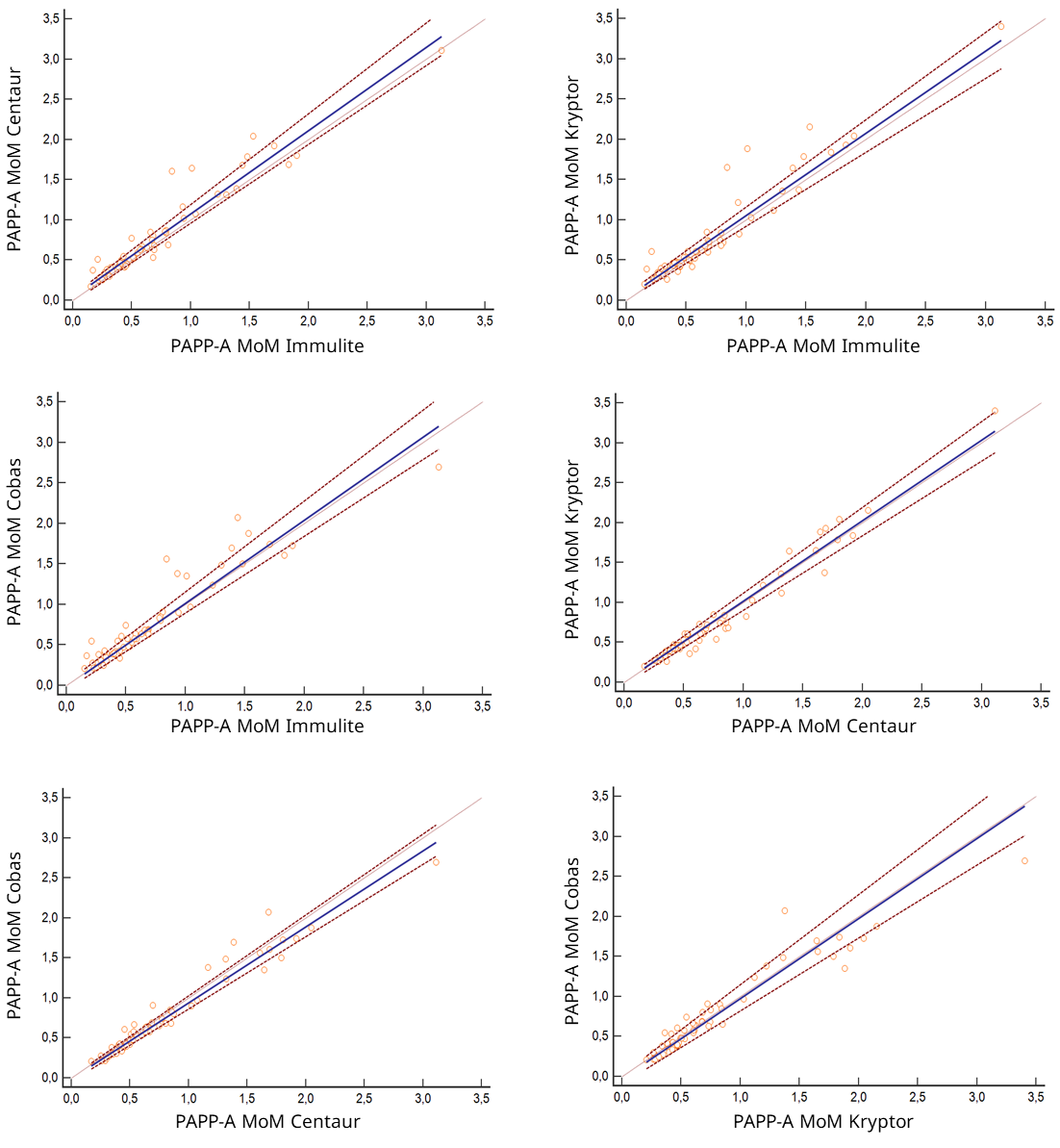

Supplement: Supplementary file 6 — Supplementary Material [file j_almed-2025-0008_suppl_006.docx]

**Supplemental Figure 7.** Bland-Altman comparison scatter diagrams for free β-hCG MoM


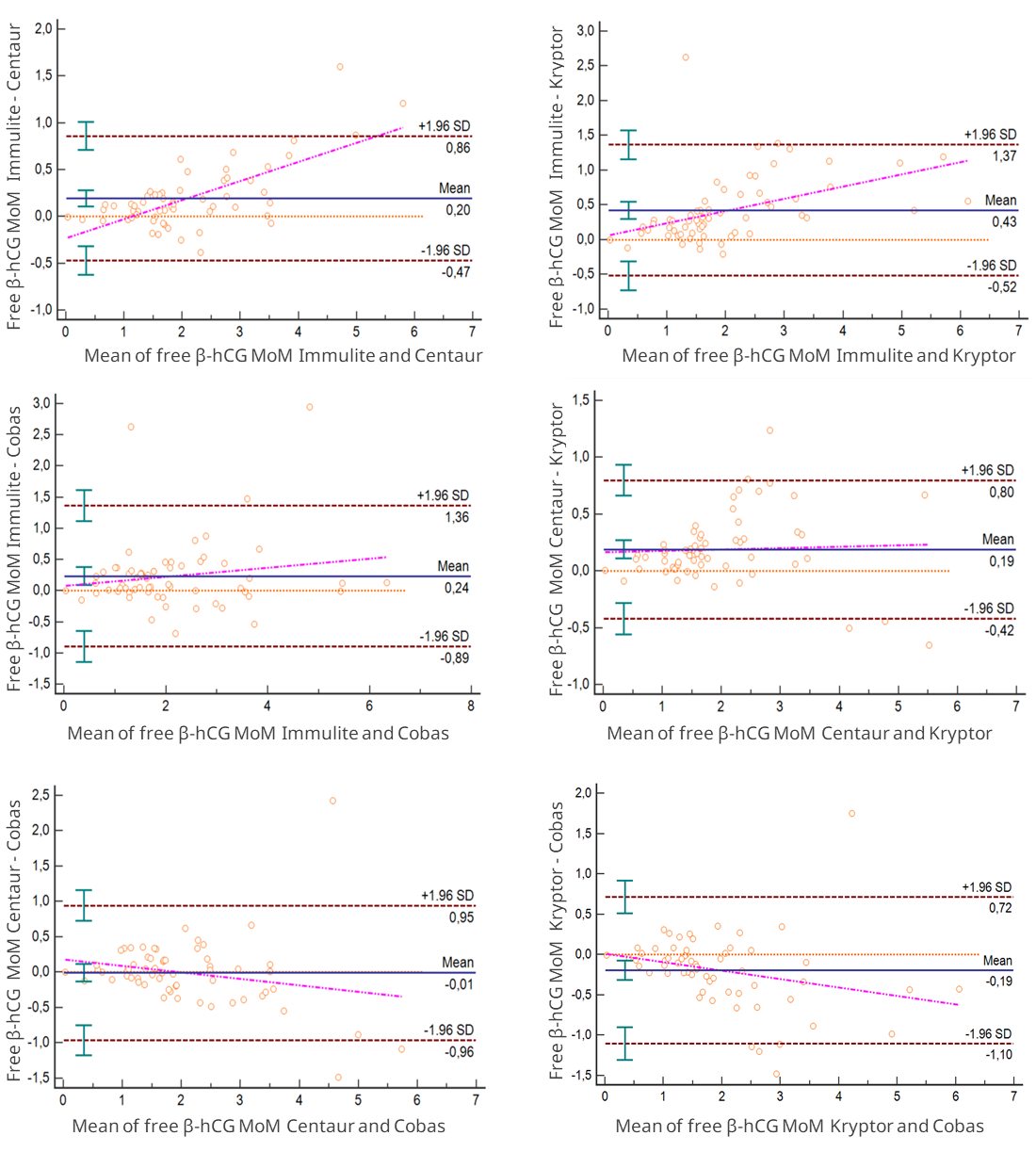

Supplement: Supplementary file 7 — Supplementary Material [file j_almed-2025-0008_suppl_007.docx]

**Supplemental Figure 8.** Bland-Altman comparison scatter diagrams for PAPP-A MoM


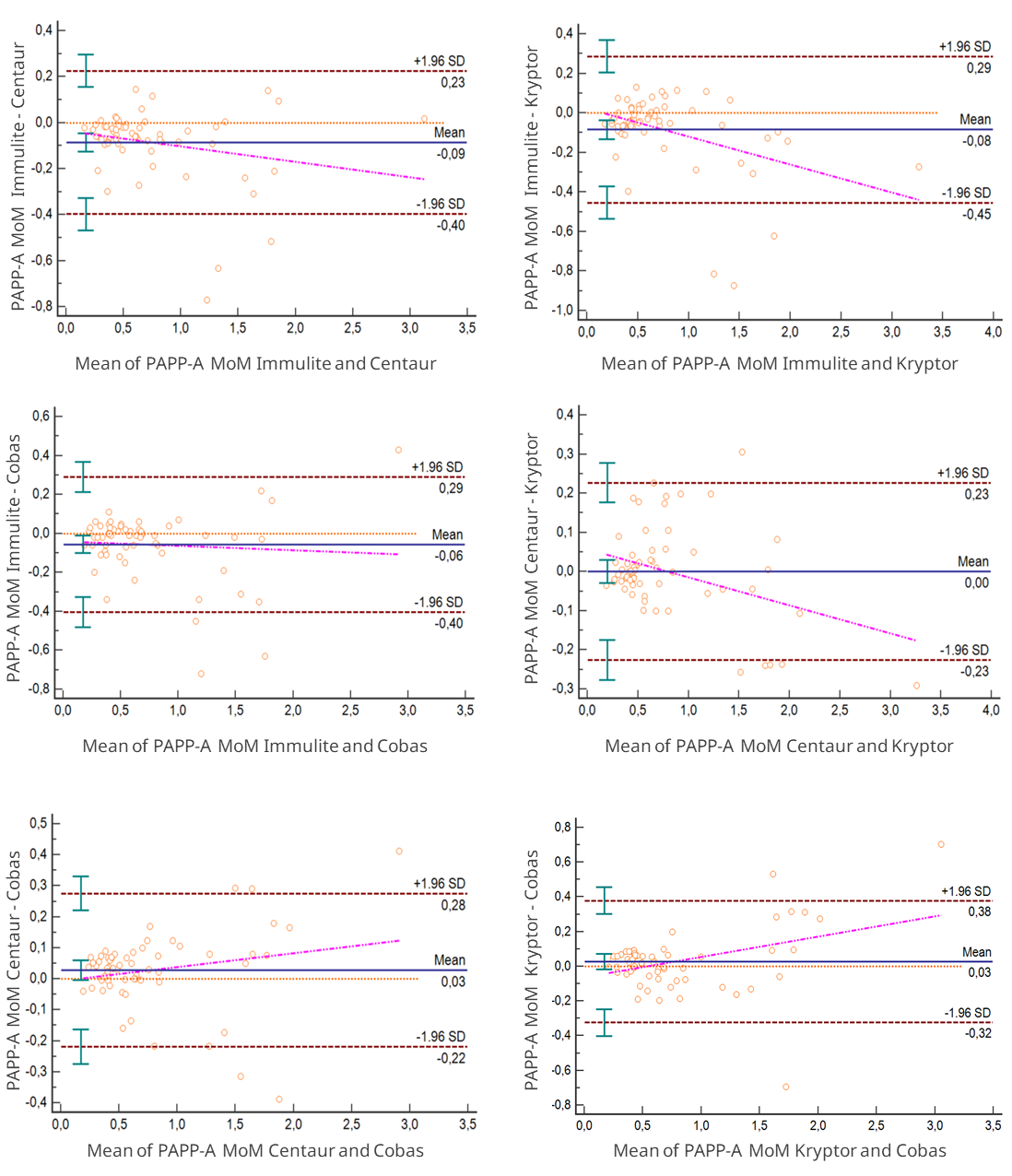

Supplement: Supplementary file 8 — Supplementary Material [file j_almed-2025-0008_suppl_008.docx]
